# Prevalence of Diagnosed Alzheimer’s Disease and Related Dementias in Medicare Advantage

**DOI:** 10.1093/geroni/igaa057.190

**Published:** 2020-12-16

**Authors:** Eric Jutkowitz, Julie Bynum, Susan Mitchell, Noelle Cocoros, Oren Shapira, Richard Platt, Ellen McCarthy

**Affiliations:** 1 Brown University, Providence, Rhode Island, United States; 2 University of Michigan, Ann Arbor, Michigan, United States; 3 Hinda and Arthur Marcus Institute for Aging Research, Boston, Massachusetts, United States; 4 Harvard Medical School Department of Population Medicine, Boston, United States; 5 Harvard Pilgrim, Boston, United States; 6 Harvardpilgrim, Boston, United States; 7 Hebrew SeniorLife, Harvard Medical School, roslindale, Massachusetts, United States

## Abstract

A third of Medicare beneficiaries are enrolled in Medicare Advantage (MA); however, little is known about MA beneficiaries diagnosed with Alzheimer’s disease and related dementias (AD/ADRD). MA plans have incentives that may influence the type of beneficiaries who enroll/disenroll from plans and the documentation of diagnoses. We calculated the prevalence of diagnosed AD/ADRD in 2014 and 2016 in three MA plans representing ~30% of the MA market. We identified beneficiaries ≥65 years of age enrolled in the MA plans in 2014 and 2016. Among eligible beneficiaries, we identified individuals with AD/ADRD using ICD-9 (2014) and ICD-10 (2016) codes included in the Medicare Chronic Conditions Warehouse algorithms for AD/ADRD. We determined the age and sex of beneficiaries diagnosed with AD/ADRD, and whether they disenrolled from the MA plan for any reason (e.g., death, enrollment in a different MA plan, enrollment in traditional Medicare or discontinuation of a plan) within 364 days from the date they were first identified has having AD/ADRD (i.e., index date). In 2014 and 2016 the prevalence of AD/ADRD diagnoses was 5.7% and 6.5%, respectively. In 2016, AD/ADRD beneficiaries were on average 82.4 (SD=7.3) years of age, 61.8% female, and had multiple comorbidities. By 364 days post-index, 32% of beneficiaries with diagnosed AD/ADRD had disenrolled from their plan. The characteristics of 2014 beneficiaries with diagnosed AD/ADRD were similar to their 2016 counterparts. In conclusion, MA beneficiaries with AD/ADRD are predominately female, have multimorbidity, and the age-stratified prevalence of AD/ADRD diagnoses is lower than rates reported in traditional Medicare.

